# Early Childhood Exposures to Fluorides and Cognitive Neurodevelopment: A Population-Based Longitudinal Study

**DOI:** 10.1177/00220345241299352

**Published:** 2024-12-18

**Authors:** L.G. Do, A. Sawyer, A. John Spencer, S. Leary, J.K. Kuring, A.L. Jones, T. Le, C.E. Reece, D.H. Ha

**Affiliations:** 1 School of Dentistry, Faculty of Health and Behavioural Sciences, The University of Queensland, Australia; 2 School of Psychology, The University of Adelaide, Australia; 3 Adelaide Dental School, The University of Adelaide, Australia; 4 Bristol Dental School, University of Bristol, UK; 5 Sunshine Coast Health Institute, Sunshine Coast University Hospital, Australia

**Keywords:** fluoride use, water fluoridation, intelligence quotient, child development, population programs, risk assessment

## Abstract

It is important to maintain confidence in the risk and benefit balance of major caries-preventive programs using fluoride. The ongoing debate about potential effects of early-life exposures to fluoride on cognitive neurodevelopment requires high-quality scientific evidence. This study aimed to investigate the potential effects of fluoride exposure on cognitive neurodevelopment assessed with the Wechsler Adult Intelligence Scale 4th edition (WAIS-IV) in an Australian population-based sample. The sample was selected from the National Child Oral Health Study (NCOHS) 2012–2014. NCOHS collected data on socioeconomic factors, oral health behaviors, and residential history to estimate percentage lifetime exposure to fluoridated water during the first 5 y of life (%LEFW). NCOHS children were also examined by trained and calibrated examiners to assess dental fluorosis (a reliable and valid individual biomarker of total fluoride intake during early childhood). The sample was followed up in 2022–2023 to collect data on cognitive neurodevelopment (intelligence quotient [IQ]) using the WAIS-IV, which was administered by trained and calibrated qualified psychologists. Multivariable regression models were generated to investigate associations between the 2 exposure measurements (%LEFW and dental fluorosis) with full-scale IQ (FSIQ) scores, controlling for important confounding effects. Hypotheses of noninferiority were also tested, contrasting different levels of exposure to fluoride. Some 357 participants aged 16 to 26 y completed the WAIS-IV, with a mean FSIQ score of 109.2 (95% confidence interval [CI]: 107.8–110.5). The estimates of the multivariable regression models demonstrated slightly higher FSIQ scores among the exposed than the nonexposed. The adjusted β of 100%LEFW versus 0%LEFW was 1.07 (95% CI: −2.86, 5.01) and of having dental fluorosis versus no fluorosis was 0.28 (95% CI: −3.00, 3.57). The hypothesis of noninferiority tests found that FSIQ scores of those exposed and nonexposed to fluoride were equivalent. The study provided consistent evidence that early childhood exposure to fluoride does not have effects on cognitive neurodevelopment.

## Introduction

Water fluoridation (WF) and fluoridated toothpaste are the main preventive programs against dental caries (McDonagh et al. 2000; Centers for Disease Control and Prevention 2001; National Health and Medical Research Council 2017). It is crucial to assure the effectiveness and safety of fluoride to ensure population health and thus to maintain public support for this important public health program. As a public health measure, preventive programs using fluoride require ongoing research to maintain its balance of risk and benefits (Do and Spencer 2007).

The developing human brain is more susceptible to changes caused by chemicals than is the mature brain. The major windows of developmental vulnerability occur during infancy and early childhood (Rice and Barone 2000). Therefore, there are concerns built on the precautionary principle that the developing human brain may be influenced by early excessive exposure to fluoride. Reports from the National Toxicology Program (NTP) raised concern that fluoride was a cognitive neurodevelopmental hazard (NTP 2020). However, that statement has been criticized for lack of credible evidence (National Academies of Sciences, Engineering, and Medicine 2020), and was later withdrawn (NTP 2024).

The topic of potential neurodevelopmental effects of fluoride has been contentiously debated. Major reviews have concluded that fluoride at the level practiced in WF programs does not negatively affect child neurodevelopment (Guth et al. 2020; Aggeborn and Öhman 2021; Kumar et al. 2023). However, some other reviews continued to raise the concern (Grandjean 2019; Grandjean et al. 2024), mostly using data from the MIREC study series (Green et al. 2019, 2020; Till et al. 2020). A recent critical appraisal of the evidence emphasized the importance of exposure measurement, outcome measurement, and analytical approaches in investigating potential effects of fluoride on cognitive neurodevelopment (Guichon et al. 2024).

This study aimed to investigate potential links between early exposure to fluoride (measured by percentage lifetime exposure to fluoridated water and the presence of dental fluorosis) and cognitive neurodevelopment (measured as full-scale intelligence quotient [IQ] using the Wechsler Adult Intelligence Scale [WAIS-IV]) in a population-based sample of Australian young adults.

## Methods

The study has received ethical approval from the University of Adelaide and University of Queensland Human Research Ethics Committees. The study follows the STROBE Guidelines for observational human research. Participants provided written informed consent at baseline and follow-up.

This study was built on a large existing population-based study, the Australia’s National Child Oral Health Study (NCOHS) 2012–2014 (Do and Spencer 2016). The NCOHS sample was selected using a multistage, stratified random selection process to ensure its population representativeness. Data collection included comprehensive parental questionnaires and a detailed oral epidemiological examination.

NCOHS children were examined by trained and calibrated examiners to record dental fluorosis (a reliable and valid clinical biomarker of total fluoride intake during early childhood). The questionnaire collected detailed information on parental socioeconomic status and child health behaviors and practices, including toothbrushing with fluoridated toothpaste and additional forms of fluoride. NCOHS data have been used to investigate the effects of fluoridation on dental caries (Spencer et al. 2018) and on socioeconomic inequality in child oral health (Do et al. 2018).

In 2020–2021, a first follow-up of the NCOHS sample investigated associations between exposure to fluoridation and child behavioral development (Do et al. 2023). As part of this follow-up study, in 2022–2023 a subset of NCOHS participants who had turned 16+ y was recruited and completed the WAIS-IV to investigate the associations between early-life exposures to fluoride and neurodevelopment (Appendix Fig. 1). Those who were identified as having dental fluorosis in the NCOHS clinical assessment were first invited to participate in the intelligence assessment. A random sample of participants without dental fluorosis with similar age (±1 y) and same sex were subsequently selected and invited, with a ratio of 4 to 1 (the prevalence of dental fluorosis was 18%). All IQ tests were conducted one-on-one in psychological clinics or specially hired quiet offices to ensure appropriate psychological test administration conditions.

### Exposure Measurements

The NCOHS questionnaire collected a detailed residential history from birth to the time of the survey and included consumption of public water and other water sources. The residential history of participants has been linked to the postcode-level fluoride concentration in public water database that was updated yearly by water authorities. The F levels in water were coded 0.5 for levels of >0.3 to ≤0.7 mgF/L, and coded 1 for levels of >0.7 to 1.1 mgF/L. The number of years residing at each location was multiplied by the fluoride concentration. The products were summed and divided by the child’s age and multiplied by 100 to express the individual-level percentage lifetime access to the equivalent of ≥0.7 mg F in drinking water (%LEFW). If fluoride-removing water filters were used at a location, or nonpublic water sources (bottled, well water, rainwater), the fluoride concentration was set to 0 mg/L. Three groups were formed: having 0%LEFW, >0% to <100%LEFW, and 100%LEFW. This is the most robust and rigorous method of assessing exposure to fluoridated water (Do et al. 2009; Slade et al. 1995) and has been well accepted internationally (Hillier et al. 2000). We used %LEFW from birth to age 5 y to maintain consistency between participants.

NCOHS children were examined by trained and calibrated dental examiners who were not aware of questionnaire results. Dental fluorosis was assessed on permanent maxillary central incisors, which develop during the first years of life. Dental fluorosis is an objective clinical biomarker of total exposure and individual susceptibility to fluoride. Examiners were trained in differentiating dental fluorosis from nonfluorotic opacities using the Russell criteria (Russell 1961). Severity of dental fluorosis was assessed using the Thylstrup and Fejerskov (TF) Index (Fejerskov et al. 1988). Most observed cases with fluorosis had TF scores of 1 or 2 (very mild to mild). Only 0.9% of the children had a TF score of 3+ (Do and Spencer 2016). For this study, participants were grouped as having (TF 1+) or not having dental fluorosis.

### Primary Outcome: Cognitive Neurodevelopment

The WAIS-IV is an individually administered standardized assessment of intelligence for people aged 16+ y. The scales consist of 10 subtests that are used to calculate the full-scale IQ (FSIQ) score and 4 indices (verbal comprehension, perceptual reasoning, working memory, and processing speed) (Wechsler 2008). Scores on these scales are age standardized and have a mean of 100 and standard deviation of 15. The WAIS-IV provides a reliable and valid estimate of intellectual functioning with high stability (Saklofske et al. 2018).

Qualified psychologists, employed to administer the WAIS-IV, underwent training and administration compliance testing ran by J.K.K., a neuropsychologist with extensive experience in Wechsler intelligence scales. All participants were administered the WAIS-IV test one-on-one with a trained psychologist in standardized conditions for the psychological assessment (in a quiet room, with participants facing a monotonic wall without any distractions). The psychologists and participants were not aware of the exposure status of the participants. Test scoring was conducted using Q-Global, which is Pearson’s web-based system for scoring and reporting assessments, including the WAIS-IV. Using the Q-Global system allowed standardized scoring and interpretation across all administering psychologists. J.K.K. regularly checked test administration and scoring of all examiners to check for any issues with assessment and scoring. The computed FSIQ scores and index scores were combined and merged with participants’ existing NCOHS data to allow for data management and analysis.

### Covariates

Covariates used in the multivariable regression models were selected as potentially influencing the associations between fluoride exposures and the outcomes (see the directed acyclic graph in Appendix Fig. 2). Covariates were age at follow-up, sex, household income, parental education and country of birth, participants’ neurodevelopmental diagnosis (attention-deficit hyperactivity disorder, autism spectrum disorder, dyslexia, dyscalculia), breastfeeding duration (never, up to 6 mo, 6 to 24 mo, and 24+ mo), and toothbrushing with fluoride toothpaste collected retrospectively for age 2 y (<2 times/day, 2+ times/day).

### Analytical Approaches

We analyzed the associations between FSIQ scores and the exposures sequentially from bivariate to multivariable generalized linear regression models using SAS Proc GENMOD. Two sets of models were generated for each exposure. Model 1 controlled for socioeconomic factors and neurodevelopmental diagnosis. Model 2 additionally controlled for breastfeeding duration and toothbrushing with fluoride toothpaste during early childhood.

We further tested hypotheses of equivalence between the groups by exposure to fluoride following standard approaches (Fleming 2008; Scott 2009; Schumi and Wittes 2011). We hypothesized that measures of cognitive neurodevelopment associated with fluoride exposure in early childhood would be noninferior to those associated with no exposure to fluoride by more than a clinically acceptable equivalence margin Δ (Fleming 2008; Scott 2009). The hypothesis of equivalence was a more appropriate approach because the traditional null hypothesis of no difference cannot provide proof of similarity (Scott 2009; Schumi and Wittes 2011). This is an important principle in evaluating the risk-benefit profiles of public health programs (Barker et al. 2002). We defined the equivalence margin Δ for the FSIQ score as one-quarter of its standard deviation (equal to 3.75).

We used SAS Proc POWER to estimate a required sample to test a hypothesis of noninferiority in FSIQ scores between children with and without exposures to fluoride (expected minimum ratios of 1 to 4) with a stringent equivalence margin Δ of 3.75 FSIQ points. Some 342 children would be required at power of 0.9, ∝ = 0.05.

We also conducted sensitivity analysis using stratified analyses by key socioeconomic factors and neurodevelopmental diagnosis (see Appendix). Multivariable models were also generated for the WAIS-IV index scores (see Appendix). While the main analyses were conducted without imputation for missing data, multiple imputation for missing data of covariates was generated as sensitivity analysis (see Appendix).

## Results

A total of 357 participants completed the WAIS-IV (Table 1). The participants’ ages ranged from 16 to 26 y, with a mean of 19.6 y (SD = 2.3) and a median of 19.4 y (IQR = 17.7–21.1). The groups by exposure to fluoridated water (%LEFW) varied by the distributions of sex, socioeconomic factors, and residential location. However, the 95% confidence intervals (CIs) of the estimates overlap. The proportions of participants who reportedly did not have a neurodevelopmental diagnosis were comparable between the 3 LEFW groups. The distributions by sex and socioeconomic factors were comparable between those with and without dental fluorosis (Table 2). A higher proportion of participants with dental fluorosis had parents born overseas. There was also a higher proportion of participants without dental fluorosis who reportedly had a neurodevelopmental diagnosis. However, the 95% CIs of the estimates overlap.

**Table 1.** Study Sample Characteristics by Percentage Lifetime Exposure to Fluoridated Water during the First 5 y of Life (*N* = 357).

	% Lifetime Exposure to Fluoridated Water (% LEFW), col % (95% CI)		
	0% (*n* = 68)	>0% to <100% (*n* = 83)	100% (*n* = 194)
Age, y, mean (SD)	20.9 (2.6)	19.6 (2.3)	19.2 (2.2)
Sex			
Male	41.2 (29.4, 52.9)	53.8 (42.9, 64.3)	47.4 (40.4, 54.5)
Female	58.8 (47.1, 70.6)	46.4 (35.7, 57.1)	52.6 (45.5, 59.6)
Parental education			
School only	9.0 (2.1, 15.8)	6.0 (0.9, 11.0)	3.1 (0.6, 5.5)
Vocational	13.4 (5.2, 21.6)	7.1 (1.6, 12.7)	10.8 (6.4, 15.2)
Tertiary	77.6 (67.6, 87.6)	86.9 (79.7, 94.2)	86.1 (81.2, 91.0)
Household income			
Low	20.6 (10.6, 30.7)	17.5 (9.1, 25.9)	15.3 (10.1, 20.4)
Medium	47.6 (35.2, 60.0)	41.3 (30.4, 52.1)	34.7 (27.9, 41.5)
High	31.7 (20.2, 43.3)	41.3 (30.4, 52.1)	50.0 (42.9, 57.1)
Parent country of birth			
Other	32.8 (21.5, 44.1)	64.3 (54.0, 74.6)	38.3 (31.4, 45.2)
Australia	67.2 (55.9, 78.5)	35.7 (25.4, 46.0)	61.7 (54.8, 68.6)
Residential location			
Regional	26.5 (15.9, 37.0)	19.0 (10.6, 27.5)	11.9 (7.3, 16.4)
Major city	73.5 (63.0, 84.1)	81.0 (72.5, 89.4)	88.1 (83.6, 92.7)
Neurodevelopmental diagnosis			
Yes	13.2 (5.1, 21.3)	8.3 (2.4, 14.3)	8.2 (4.4, 12.1)
No	73.5 (63.0, 84.1)	76.2 (67.0, 85.3)	75.8 (69.7, 81.8)
Not reported	13.2 (5.1, 21.3)	15.5 (7.7, 23.2)	16.0 (0.8, 21.2)
Breastfeeding duration			
Never breastfed	12.3 (4.3, 20.3)	10.7 (4.1, 17.4)	12.6 (7.9, 17.4)
Breastfed to <6 mo	27.7 (16.8, 38.6)	41.7 (31.1, 52.3)	26.3 (20.0, 32.6)
Breastfed 6 to 24 mo	43.1 (31.0, 55.2)	42.9 (32.2, 53.5)	52.6 (45.5, 59.8)
Breastfed 24+ mo	16.9 (7.8, 26.1)	4.8 (0.2, 9.3)	8.4 (4.5, 12.4)
Toothbrushing frequency			
<2 times/day	40.3 (28.5, 52.1)	39.0 (28.4, 49.6)	45.7 (38.3, 53.1)
2+ times/day	59.7 (47.9, 71.5)	61.0 (50.4, 71.6)	54.3 (46.9, 61.7)

95% CI, 95% confidence intervals of the estimates; % LEFW, percentage lifetime exposure to fluoridated water.

Study sample: the National Child Oral Health Study 2012–2014 sample who were aged 16+ y and have completed an IQ test for the follow-up 2022–2023. Twelve participants had missing data on %LEFW. Neurodevelopmental diagnosis: study participants who reportedly had at least 1 diagnosed condition (attention-deficit hyperactivity disorder, autism spectrum disorder, dyslexia, dyscalculia). Toothbrushing frequency: toothbrushing with fluoride toothpaste at the age of 2 y.

**Table 2.** Study Sample Characteristics by Presence of Dental Fluorosis on Maxillary Central Incisors (*N* = 357).

	Dental fluorosis, col % (95% CI)	
	No (*n* = 256)	Yes (*n* = 83)
Age, y, mean (SD)	19.6 (2.3)	19.5 (2.2)
Sex		
Male	47.3 (41.1, 53.4)	41.7 (31.1, 52.3)
Female	52.7 (46.6, 58.9)	58.3 (47.7, 68.9)
Parental education		
School only	5.6 (2.7, 8.4)	4.9 (0.2, 9.7)
Vocational	9.9 (6.2, 13.6)	12.3 (5.1, 19.5)
Tertiary	84.5 (80.0, 89.0)	82.7 (74.4, 91.0)
Household income		
Low	18.3 (13.4, 23.1)	14.6 (6.9, 22.3)
Medium	38.2 (32.1, 44.3)	40.2 (29.6, 50.9)
High	43.5 (37.3, 49.7)	45.1 (34.3, 55.9)
Parent country of birth		
Other	45.3 (39.1, 51.4)	34.9 (24.6, 45.2)
Australia	54.7 (48.6, 60.9)	65.1 (54.8, 75.4)
Residential location		
Regional	15.2 (10.8, 19.7)	23.8 (14.7, 33.0)
Major city	84.8 (80.3, 89.2)	76.2 (67.0, 85.3)
Neurodevelopmental diagnosis		
Yes	9.8 (6.1, 13.4)	6.0 (0.9, 11.0)
No	76.2 (70.9, 81.4)	73.8 (64.4, 83.3)
Not reported	14.1 (9.8, 18.3)	20.2 (11.6, 28.9)
Breastfeeding duration		
Never breastfed	11.5 (7.5, 15.5)	14.8 (7.0, 22.6)
Breastfed to <6 mo	30.6 (24.8, 36.3)	29.6 (19.6, 39.6)
Breastfed 6 to 24 mo	48.4 (42.2, 54.6)	49.4 (38.4, 60.3)
Breastfed 24+ mo	9.5 (5.9, 13.2)	6.2 (0.9, 11.4)
Toothbrushing frequency		
<2 times/day	43.8 (37.4, 50.1)	38.0 (27.2, 48.7)
2+ times/day	56.3 (49.9, 62.6)	62.0 (51.3, 72.8)

95% CI, 95% confidence intervals of estimates; SD, standard deviation.

Study sample: the National Child Oral Health Study 2012–2014 sample who were aged 16+ y and have completed an IQ test for the follow-up 2022–2023. Sixteen participants had missing data on dental fluorosis. Neurodevelopmental diagnosis: study participants who reportedly had at least 1 diagnosed condition (attention-deficit hyperactivity disorder, autism spectrum disorder, dyslexia, dyscalculia). Toothbrushing frequency: frequency of toothbrushing with fluoride toothpaste at the age of 2 y per day.

The study sample had a mean full-scale IQ score of 109.2 (95% CI: 107.8–110.5). Participants who had 0%LEFW had a slightly lower unadjusted mean full-scale IQ score than the other 2 exposure groups did (Table 3). The multivariable models 1 and 2 demonstrated that those who had 0%LEFW had lower FSIQ scores than those who had whole or part of their first 5 y of life exposed to fluoridated water. The largest difference was found between the >0% to <100%LEFW group and the 0%LEFW group.

**Table 3.** Unadjusted Full-Scale IQ Scores by Exposures and Multivariable Regression Models for Adjusted Full-Scale IQ Scores by Percentage Lifetime Exposure to Fluoridated Water.

	Full-Scale IQ Score (*N* = 357)	Multivariable Model 1	Multivariable Model 2
	Mean (95% CI)	β (95% CI)	β (95% CI)
Model intercept		109.4 (105.3, 113.5)	110.7 (106.3, 115.2)
Lifetime exposure to fluoridated water			
100%	109.1 (107.3, 110.9)	1.12 (−2.81, 5.05)	1.07 (−2.86, 5.01)
>0% to <100%	110.7 (107.6, 113.7)	2.24 (−2.17, 6.66)	2.52 (−1.92, 7.00)
0%	108.6 (105.6, 111.5)	Ref	Ref

95% CI, 95% confidence intervals of estimates; Ref, reference.

Study sample: the National Child Oral Health Study 2012–2014 sample who were aged 16+ y and have completed an IQ test for the follow-up 2022–2023. Neurodevelopmental diagnosis: study participants who reportedly had at least 1 diagnosed condition (attention-deficit hyperactivity disorder, autism spectrum disorder, dyslexia, dyscalculia). Toothbrushing frequency: frequency of toothbrushing with fluoride toothpaste at the age of 2 y per day. Estimates of covariates are presented in Appendix Table 1. Model 1: multivariable regression models controlling for socioeconomic factors, neurodevelopmental diagnosis, and age at IQ test. Model 2: model 1 plus covariates (breastfeeding duration and toothbrushing frequency at age 2 y).

The unadjusted mean FSIQ scores were comparable between those with or without dental fluorosis on their maxillary central incisors (Table 4). Both multivariable models also confirmed that those 2 groups did not have meaningful differences in their FSIQ scores. Estimates of other coefficients from the same models are presented in the Appendix (Appendix Tables 1 and 2).

**Table 4.** Unadjusted Full-Scale IQ Scores by Exposures and Multivariable Regression Models for Adjusted Full-Scale IQ Scores by Dental Fluorosis.

	Full-Scale IQ Score (*N* = 357)	Multivariable Model 1	Multivariable Model 2
	Mean (95% CI)	β (95% CI)	β (95% CI)
Model intercept		110.2 (107.3, 113.1)	111.6 (108.3, 114.9)
Dental fluorosis			
Yes	109.4 (107.8, 111.0)	0.27 (−3.04, 3.58)	0.28 (−3.00, 3.57)
No	109.3 (106.5, 112.1)	Ref	Ref

95% CI, 95% confidence intervals of estimates; Ref, reference.

Study sample: the National Child Oral Health Study 2012–2014 sample who were aged 16+ y and have completed an IQ test for the follow-up 2022–2023. Neurodevelopmental diagnosis: study participants who reportedly had at least 1 diagnosed condition (attention-deficit hyperactivity disorder, autism spectrum disorder, dyslexia, dyscalculia). Toothbrushing frequency: frequency of toothbrushing with fluoride toothpaste at the age of 2 y per day. Estimates of covariates are presented in Appendix Table 2. Model 1: multivariable regression models controlling for socioeconomic factors, neurodevelopmental diagnosis, and age at IQ test. Model 2: model 1 plus covariates (breastfeeding duration and toothbrushing frequency at age 2 y).

**Figure 1.**
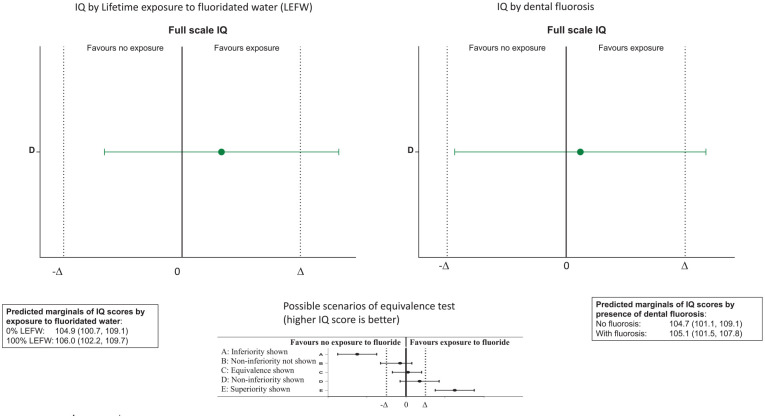
Equivalence test of IQ scores by percentage lifetime exposure to fluoridated water and by presence of dental fluorosis.

Equivalence tests were conducted for full model-adjusted IQ scores between those with 100%LEFW versus 0%LEFW and between those with versus those without dental fluorosis (Fig. 1). The adjusted FSIQ scores of those who had 100%LEFW were slightly higher (better) than those of the 0%LEFW group. The lower 95% CI bound of the estimate was higher than the lower bound of the equivalence margins, whereas the upper 95% CI of the estimate was higher than the upper bound of the equivalence margin. This indicates that the FSIQ scores of those who had 100%LEFW were not inferior to the FSIQ scores of the 0%LEFW group. Similarly, the adjusted estimate of the FSIQ score of those with dental fluorosis was not inferior to that of those without dental fluorosis. The 95% CIs of the estimate were also slightly higher than the lower and upper bounds of the equivalence margins.

Similar multivariable models stratified by sex, parental education, household income, and neurodevelopmental diagnosis demonstrated that the full-scale IQ scores were comparable across different levels of exposure to fluoride in early childhood in this study sample (Appendix Tables 3–6). Similarly, multivariable models generated for WAIS-IV index scores (Verbal Comprehension Index, Perceptual Reasoning Index, Working Memory Index, Processing Speed Index) demonstrated no associations between different levels of fluoride exposure with those index scores (Appendix Table 7). Multiple imputation analysis did not reveal any associations between %LEFW and dental fluorosis with the FSIQ score (Appendix Tables 8–10).

## Discussion

This multidisciplinary follow-up study investigated if early-life exposure to fluoride (measured by exposure to fluoridated water during the first 5 y of life and presence of dental fluorosis) had an effect on child cognitive neurodevelopment (IQ scores measured by the WAIS-IV). The multiple comprehensive approaches used in the study have consistently demonstrated that early-life exposure to fluoride by Australian children did not have any measurable effect on their cognitive neurodevelopment.

The findings of the study are in line with recent major systematic reviews (Canadian Agency for Drugs and Technologies in Health 2020; Guth et al. 2020; Aggeborn and Öhman 2021; Kumar et al. 2023; Veneri et al. 2023) and recent individual studies (Ibarluzea et al. 2022; Lin et al. 2023). These reviews summarized epidemiological evidence of the potential association between exposure to fluoride and cognitive neurodevelopment. These reviews concluded that exposure to fluoride at the levels practiced in community WF programs was not associated with a negative effect on cognitive neurodevelopment. The reported negative association between fluoride exposure and IQ was observed in some included studies with high risk of bias but not in studies with low risk of bias (Veneri et al. 2023). Such findings emphasized the importance of quality investigation including exposure measurement, outcome measurement, and controlling for potential confounding effects.

This study used 2 different measures of fluoride exposure in early childhood to ensure comprehensiveness in exposure measurement. The %LEFW has been frequently used to investigate the effect of WF on dental caries and dental fluorosis in Australian studies (Do and Spencer 2007; Slade et al. 2018; Spencer et al. 2018). This measure is an objective quantification of the amount of lifetime a person resided in an area, the fluoride level of public water supplies, and use of water during that life period. We maximized the validity of %LEFW by also considering sources of water and water filtration. The focus on the 0- 5-y age period further standardized the exposure. This exposure measurement was used to investigate the association with dental caries in the same population (Spencer et al. 2018). A strong and dose-response association was reported, indicating that %LEFW actually measured fluoride exposure in this population, given the primary action of fluoride in preventing dental caries. Dental fluorosis is a reliable and valid individual biological marker of sustained total intake of fluoride in early childhood (Fejerskov et al. 1988). This measure is an individual indicator of actual total intake of fluoride during a certain life period (the first 3 y of life for maxillary central incisors) and can be measured only through clinical assessment, which was assessed by trained and calibrated dental examiners (Do and Spencer 2016). To prevent unconscious bias, we ensured that the study psychologists were blinded of the exposure status of the participants.

In this current study sample, analysis using both exposure measures consistently indicated that early-life exposure to fluoride had no effect on cognitive development. A recent study investigating the association between dental fluorosis and IQ in children aged 6 to 12 y (Lin et al. 2023) also reported no association between dental fluorosis and IQ, similar to our findings.

The primary outcome measurement, IQ, is a complex measure to collect (Nisbett et al. 2012). Variability in collecting IQ data may lead to incorrect findings (Guichon et al. 2024). We focused on training and calibrating WAIS-IV administrators who were already qualified psychologists to achieve high quality and consistency of assessment. The chief examiner (J.K.K.) conducted training and administration compliance testing with all examiners to ensure accurate and consistent administration and scoring across the team. We used the WAIS-IV to collect IQ data, which has long been considered the gold standard of intelligence assessment. The test’s full set of index scores allows for a comprehensive evaluation of different components of cognitive development. While the use of the WAIS-IV was time-consuming and costly, its comprehensiveness provided sufficient quality of data to address our important research question. The excellent stability of the WAIS-IV in measuring IQ in adults ensured the accuracy of the outcome as compared with other instruments used in children (Saklofske et al. 2018). We also analyzed WAIS-IV index scores as secondary outcome variables. While increasing the number of analyses might lead to false-positive findings, the lack of such errors further supported our overall conclusion.

Further to having reliable and valid exposure measurements and outcomes, our analytical approaches aimed to reduce potential analytical biases. We developed our analytical models using a theory-based approach. The variables included in the models were selected a priori with a directed acyclic graph to control for confounding effects. We also included breastfeeding duration, a strong factor associated with cognitive neurodevelopment (Mortensen et al. 2002; Nisbett et al. 2012). Toothbrushing with fluoridated toothpaste at the age of 2 y was also included to investigate its potential effect.

Another important feature of the study was the testing of a hypothesis of equivalence. Such a feature is important in addressing the question of whether or not early-life exposure to fluoride alters normal cognitive neurodevelopment. Under the strict criteria of equivalence hypothesis testing, and stringent equivalence margins, our analyses demonstrated that IQ scores of those who had early-life exposure to fluoride were at least equivalent to those without exposure. The evidence of equivalence was observed even between the opposite extreme groups in terms of exposure to fluoride. In other words, it can be concluded with confidence that early-life exposure to fluoride did not alter normal cognitive neurodevelopment in this study population.

A potential limitation of the study was the relatively smaller sample size than the original NCOHS sample. However, this sample has been estimated to have adequate power to test the hypothesis of equivalence using stringent equivalence margins. The sample was also randomly selected from a nationally representative population. It was not possible to conduct the IQ assessments on all eligible NCOHS participants due to its very high cost and the time-consuming nature of the WAIS-IV. It could be argued that %LEFW was not a biological individual measure of exposure. However, we calculated %LEFW using individual factors to capture variations between individuals. There might be potential diffusion effects of WF; however, any such diffusion effects would likely be minor in Australia given the distribution of the population and would not meaningfully change the categorizations of exposures to WF. The use of individual-level dental fluorosis enhanced our exposure measurements. The presence and severity of dental fluorosis are sensitive to the total intake of fluoride. Dental fluorosis was mild in our study, so that possible effects of much higher levels of fluoride intake may not be observed. Our results are relevant to populations with developed programs such as WF. The unadjusted FSIQ score was slightly lower in the 0%LEFW group than in the 100%LEFW (0.5 IQ), which was within the margin of error. The FSIQ scores in the study sample were higher than the population norms, a phenomenon reported in developed countries (Nisbett et al. 2012). Finally, there were small amounts of missing data. The impact of this was examined in the sensitivity analyses with multiple imputation, which did not alter the overall conclusion.

## Conclusion

This population-based follow-up study has provided consistent scientific evidence that early-life exposure to fluoride was not negatively associated with cognitive neurodevelopment. The findings, in combination with the current body of knowledge, provide assurance that the currently practiced WF programs are both effective and safe for young children.

## Authors’ Contributions

L.G. Do, contributed to conception and design, data acquisition, analysis, and interpretation, drafted and critically revised the manuscript; A. Sawyer, contributed to conception and design, data acquisition and interpretation, critically revised manuscript; A. John Spencer, S. Leary, A. Jones, contributed to conception and design, data interpretation, critically revised manuscript; J.K. Kuring, T. Le, C.E. Reece, contributed to design, data acquisition and interpretation, critically revised the manuscript; D.H. Ha, contributed to conception and design, data acquisition, analysis, and interpretation, critically revised the manuscript. All authors gave their final approval and have agreed to be accountable for all aspects of the work.

## Supplemental Material

sj-docx-1-jdr-10.1177_00220345241299352 – Supplemental material for Early Childhood Exposures to Fluorides and Cognitive Neurodevelopment: A Population-Based Longitudinal Study Supplemental material, sj-docx-1-jdr-10.1177_00220345241299352 for Early Childhood Exposures to Fluorides and Cognitive Neurodevelopment: A Population-Based Longitudinal Study by L.G. Do, A. Sawyer, A. John Spencer, S. Leary, J.K. Kuring, A.L. Jones, T. Le, C.E. Reece and D.H. Ha in Journal of Dental Research
