# Supplementary material for: Early Childhood Exposures to Fluorides and Cognitive Neurodevelopment: A Population-Based Longitudinal Study
Source: J Dent Res. 2024 Dec 18;104(3):243–50. doi: 10.1177/00220345241299352 (PMC11843800; doi:10.1177/00220345241299352)
Supplement: sj-docx-1-jdr-10.1177_00220345241299352 – Supplemental material for Early Childhood Exposures to Fluorides and Cognitive Neurodevelopment: A Population-Based Longitudinal Study [file sj-docx-1-jdr-10.1177_00220345241299352.docx]

**Full title: Early childhood exposures to fluorides and cognitive neurodevelopment: A population-based longitudinal study**

**Loc G Do***, PhD, email: l.do@uq.edu.au
School of Dentistry, Faculty of Health and Behavioural Sciences, The University of Queensland, Australia

**Alyssa Sawyer**, PhD, email: Alyssa.sawyer@adelaide.edu.au
School of Psychology, The University of Adelaide, Australia

**A John Spencer**, PhD, email: john.spencer@adelaide.edu.au
Adelaide Dental School, The University of Adelaide, Australia

**Sam Leary**, PhD, email: s.d.leary@bristol.ac.uk
Bristol Dental School, University of Bristol, UK

**Julia Kate Kuring**, MClinPsych email: [julia.kuring@adelaide.edu.au](mailto:julia.kuring@adelaide.edu.au)
School of Psychology, The University of Adelaide, Australia

**Alison Jones**, PhD, email: alisonj@uow.edu.au
Faculty of Science, Medicine and Health, University of Wollongong, Australia

**Thu Le**, PhD, email: [thu.le@uq.edu.au](mailto:thu.le@uq.edu.au)
School of Dentistry, Faculty of Health and Behavioural Sciences, The University of Queensland, Australia

**Christy Elizabeth Reece**, BPsych, email: [christy.reece@adelaide.edu.au](mailto:christy.reece@adelaide.edu.au)
School of Psychology, The University of Adelaide, Australia

**Diep H Ha**, PhD, email: d.ha@uq.edu.au
School of Dentistry, Faculty of Health and Behavioural Sciences, The University of Queensland, Australia

**Corresponding author:**

Professor Loc Do,

School of Dentistry, Faculty of Health and Behavioural Sciences, The University of Queensland, Australia

Email: [l.do@uq.edu.au](mailto:l.do@uq.edu.au)

**Appendix**

METHODS

Random selection.

Matched by age and sex,
ratio 4 to 1

**Appendix Figure 1:** **Diagram of sample selection and recruitment**.

| **C**  **E**  **Y**  **Birth 2021-23**  **M** | **C (**confounders): Sex; Household income; Parental education; Family composition; Country of birth (parents); Residential locations (current and early life);  **E**: (Exposures)   - Exposure to fluoridated water from birth to 5 years - Dental fluorosis experience.   **Y** (Outcomes): FSIQ score.  **M**: (covariates):   - Breastfeeding duration - Toothbrushing with fluoride toothpaste at age two years - Neurodevelopmental diagnosis |
| --- | --- |

**Appendix Figure 2: Simplified Direct Acyclic Graph (DAG) for the Specific Aims**

RESULTS

Estimates of all covariates from the multivariable models for IQ scores are presented in Appendix Tables 1 and 2.

**Table 1:** Full scale IQ scores by exposures and characteristics and multivariable regression models for full scale IQ scores by per cent lifetime exposure to fluoridated water

|  | Full scale IQ score (n=357) | Multivariable Model 1 | Multivariable Model 2 |
| --- | --- | --- | --- |
|  | **Mean (95%CI)** | **β (95%CI)** | **β (95%CI)** |
| Model Intercept |  | 109.4 (105.3, 113.5) | 110.7 (106.3, 115.2) |
| Lifetime exposure to fluoridated water |  |  |  |
| 100% | 109.1 (107.3, 110.9) | 1.12 (-2.81, 5.05) | 1.07 (-2.86, 5.01) |
| >0 -<100% | 110.7 (107.6, 113.7) | 2.24 (-2.17, 6.66) | 2.52 (-1.92, 7.00) |
| 0% | 108.6 (105.6, 111.5) | Ref | Ref |
| Sex |  |  |  |
| Male | 108.9 (106.9, 111.0) | 0.45 (-2.43, 3.33) | 0.37 (-2.51, 3.25) |
| Female | 109.2 (107.4, 111.0) | Ref | Ref |
| Parental education |  |  |  |
| School only | 100.1 (91.7, 108.4) | -4.44 (-10.97, 2.74) | -3.18 (-10.16, 3.80) |
| Vocational | 103.6 (100.0, 107.1) | -6.66 (-11.32, -1.99) | -6.58 (-11.21, -1.94) |
| Tertiary | 110.5 (109.0, 111.9) | Ref | Ref |
| Household income |  |  |  |
| Low | 107.1 (103.4, 110.8) | -2.18 (-6.42, 2.06) | -2.17 (-6.50, 2.15) |
| Medium | 108.5 (106.4, 110.7) | -1.14 (-4.30, 2.02) | -1.14 (-4.29, 2.01) |
| High | 110.6 (108.5, 112.7) | Ref | Ref |
| Parent country of birth |  |  |  |
| Other | 111.6 (109.4, 113.7) | 2.13 (-0.87, 5.12) | 2.32 (-0.66, 5.30) |
| Australia | 107.2 (105.4, 108.9) | Ref | Ref |
| Residential location |  |  |  |
| Regional | 108.6 (104.9, 112.3) | 2.46 (-1.42, 6.34) | 2.58 (-1.30, 6.45) |
| Major city | 109.2 (107.7, 110.6) | Ref | Ref |
| Neurodevelopmental diagnosis |  |  |  |
| Yes | 104.3 (98.9, 109.7) | -3.98 (-8.93, 0.96) | -4.03 (-9.11, 1.04) |
| No | 110.0 (108.4, 111.5) | Ref | Ref |
| Not reported | 107.4 (104.1, 110.6) | -3.26 (-7.28, 0.76) | -2.92 (-6.93, 1.10) |
| Breastfeeding duration |  |  |  |
| Never breastfed | 106.5 (102.2, 110.8) | - | -2.75 (-7.32, 1.82) |
| Breastfed to <6 mos | 108.0 (105.7, 110.4) | - | -3.36 (-6.63, -0.09) |
| Breastfed 6­–24 mos | 110.4 (108.3, 112.4) | - | Ref |
| Breastfed 24+ mos | 108.7 (104.9, 112.5) | - | -0.42 (-5.54, 4.70) |
| Toothbrushing frequency |  |  |  |
| <2 times/day | 108.0 (105.9, 110.1) | - | -0.50 (-3.30, 2.31) |
| 2+ times/day | 110.0 (108.1, 111.9) | - | Ref |

95% CI: 95% confidence intervals of estimates.

Study sample: The NCOHS 2012-14 sample who were aged 16+years and have complete IQ test for the Follow-up 2022-23.

Neurodevelopmental diagnosis: Study participants who reportedly had at least one diagnosed condition (ADHD, Autism Spectrum Disorder, dyslexia, dyscalculia).
Toothbrushing frequency: Frequency of toothbrushing with fluoride toothpaste at the age of two years per day.

Model 1: Multivariable regression models controlling for socioeconomic factors, neurodevelopmental diagnosis and age at IQ test.

Model 2: Model 1 plus covariates (breastfeeding duration, and toothbrushing frequency at age two years).

Table 2: Full scale IQ scores by exposures and characteristics and multivariable regression models for full scale IQ scores by dental fluorosis

|  | Full scale IQ score (n=357) | Multivariable Model 1 | Multivariable Model 2 |
| --- | --- | --- | --- |
|  | **Mean (95%CI)** | **β (95%CI)** | **β (95%CI)** |
| Model Intercept |  | 110.2 (107.3, 113.1) | 111.6 (108.3, 114.9) |
| Dental fluorosis |  |  |  |
| Yes | 109.4 (107.8, 111.0) | 0.27 (-3.04, 3.58) | 0.28 (-3.00, 3.57) |
| No | 109.3 (106.5, 112.1) | Ref | Ref |
| Sex |  |  |  |
| Male | 108.9 (106.9, 111.0) | 0.48 (-2.43, 3.39) | 0.35 (-2.55, 3.25) |
| Female | 109.2 (107.4, 111.0) | Ref | Ref |
| Parental education |  |  |  |
| School only | 100.1 (91.7, 108.4) | -2.88 (-9.84, 4.08) | -2.06 (-9.10, 4.97) |
| Vocational | 103.6 (100.0, 107.1) | -7.26 (-11.96, -2.55) | -7.16 (-11.83, -2.48) |
| Tertiary | 110.5 (109.0, 111.9) | Ref | Ref |
| Household income |  |  |  |
| Low | 107.1 (103.4, 110.8) | -1.42 (-5.74, 2.89) | -1.27 (-5.67, 3.13) |
| Medium | 108.5 (106.4, 110.7) | -1.37 (-4.55, 1.80) | -1.30 (-4.46, 1.86) |
| High | 110.6 (108.5, 112.7) | Ref | Ref |
| Parent country of birth |  |  |  |
| Other | 111.6 (109.4, 113.7) | 2.87 (-0.89, 5.83) | 3.19 (0.23, 6.14) |
| Australia | 107.2 (105.4, 108.9) | Ref | Ref |
| Residential location |  |  |  |
| Regional | 108.6 (104.9, 112.3) | 2.53 (-1.37, 6.44) | 2.69 (-1.19, 6.57) |
| Major city | 109.2 (107.7, 110.6) | Ref | Ref |
| Neurodevelopmental diagnosis |  |  |  |
| Yes | 104.3 (98.9, 109.7) | -3.46 (-8.52, 1.61) | -3.20 (8.39, 1.99) |
| No | 110.0 (108.4, 111.5) | Ref | Ref |
| Not reported | 107.4 (104.1, 110.6) | -2.91 (-6.96, 1.14) | -2.54 (-6.58, 1.50) |
| Breastfeeding duration |  |  |  |
| Never breastfed | 106.5 (102.2, 110.8) | - | -2.73 (-7.31, 1.84) |
| Breastfed to <6 mos | 108.0 (105.7, 110.4) | - | -3.77, -7.06, -0.48) |
| Breastfed 6­–24 mos | 110.4 (108.3, 112.4) | - | Ref |
| Breastfed 24+ mos | 108.7 (104.9, 112.5) | - | -1.31 (-6.50, 3.88) |
| Toothbrushing frequency |  |  |  |
| <2 times/day | 108.0 (105.9, 110.1) | - | -0.23 (-3.08, 2.61) |
| 2+ times/day | 110.0 (108.1, 111.9) | - | Ref |

95% CI: 95% confidence intervals of estimates.

Study sample: The NCOHS 2012-14 sample who were aged 16+years and have completed IQ test for the Follow-up 2022-23.

Neurodevelopmental diagnosis: Study participants who reportedly had at least one diagnosed condition (ADHD, Autism Spectrum Disorder, dyslexia, dyscalculia).
Toothbrushing frequency: Frequency of toothbrushing with fluoride toothpaste at the age of two years per day.

Model 1: Multivariable regression models controlling for socioeconomic factors, neurodevelopmental diagnosis and age at IQ test.

Model 2: Model 1 plus covariates (breastfeeding duration, and toothbrushing frequency at age two years).

**Stratified analysis**

Similar generalised multivariable regression models were generated for the full-scale IQ scores, stratified by key determinants of the exposure and the outcomes (sex, neurodevelopmental diagnosis, household income and parental education) (Table 3 to 6).

**Table 3:** Stratified analysis by sex: Multivariable generalised linear regression models for full scale IQ scores

|  | IQ score | |
| --- | --- | --- |
| Sex | **Male** (n=165) | **Female** (n=192) |
| % Lifetime exposure to fluoridated water (% LEFW), β (95%CI) |  |  |
| 100% | 1.33 (-5.25, 7.90) | 1.68 (-3.56, 6.92) |
| >0%-<100% | 1.12 (-6.58, 8.83) | 5.74 (-0.22, 11.70) |
| 0% | Ref | Ref |
|  |  |  |
| Dental fluorosis, β (95%CI) |  |  |
| Yes | 3.10 (-1.79, 7.99) | -2.32 (-6.66, 2.02) |
| No | Ref | Ref |
|  |  |  |

Multivariable regression models controlling for socioeconomic factors, neurodevelopmental diagnosis, age at IQ test, breastfeeding duration, and toothbrushing frequency at age two years.

**Table 4:** Stratified analysis by neurodevelopmental diagnosis (Yes vs No): Multivariable generalised linear regression models for full scale IQ scores

|  | IQ score | |
| --- | --- | --- |
| Neurodevelopmental diagnosis | **Yes** (n=55) | **No** (n=270) |
| % Lifetime exposure to fluoridated water (% LEFW), β (95%CI) |  |  |
| 100% | 16.37 (5.52, 27.23) | 0.50 (-4.12, 5.13) |
| >0%-<100% | 25.83 (10.42, 41.23) | 1.58 (-3.63, 6.79) |
| 0% | Ref | Ref |
|  |  |  |
| Dental fluorosis, β (95%CI) |  |  |
| Yes | 19.60 (1.48, 37.71) | -0.33 (-4.09, 3.43) |
| No | Ref | Ref |
|  |  |  |

Multivariable regression models controlling for socioeconomic factors, neurodevelopmental diagnosis, age at IQ test, breastfeeding duration, and toothbrushing frequency at age two years.

**Table 5:** Stratified analysis by household income (Low and Medium vs High): Multivariable generalised linear regression models for full scale IQ scores

|  | Household income | |
| --- | --- | --- |
| Household income | **Low/Medium** (n=193) | **High** (n=149) |
| % Lifetime exposure to fluoridated water (% LEFW), β (95%CI) |  |  |
| 100% | 3.69 (-1.52, 8.89) | 0.81 (-5.85, 7.47) |
| >0%-<100% | 3.35 (-2.45, 9.15) | 3.73 (-3.80, 11.26) |
| 0% | Ref | Ref |
|  |  |  |
| Dental fluorosis, β (95%CI) |  |  |
| Yes | -0.97 (-5.50, 3.56) | 0.73 (-3.88, 5.33) |
| No | Ref | Ref |
|  |  |  |

Multivariable regression models controlling for socioeconomic factors, neurodevelopmental diagnosis, age at IQ test, breastfeeding duration, and toothbrushing frequency at age two years.

**Table 6:** Stratified analysis by parental education attainment (School and Vocational vs Tertiary): Multivariable generalised linear regression models for full scale IQ scores

|  | Parental education | |
| --- | --- | --- |
| Parental education | **School/Vocational** (n=56) | **Tertiary** (n=293) |
| % Lifetime exposure to fluoridated water (% LEFW), β (95%CI) |  |  |
| 100% | 0.75 (-8.15, 9.64) | 2.04 (-2.56, 6.64) |
| >0%-<100% | 8.01 (-2.50, 18.52) | 2.64 (-2.68, 7.97) |
| 0% | Ref | Ref |
|  |  |  |
| Dental fluorosis, β (95%CI) |  |  |
| Yes | 1.21 (-7.88, 10.31) | -0.04 (-3.53, 3.43) |
| No | Ref | Ref |
|  |  |  |

Multivariable regression models controlling for socioeconomic factors, neurodevelopmental diagnosis, age at IQ test, breastfeeding duration, and toothbrushing frequency at age two years.

**WAIS Index score analysis**

Generalised linear multivariable regression models generated for WAIS Index scores to investigate adjusted effect of %LEFW and dental fluorosis (Table 7).

**Table 7**: Multivariable generalised linear regression models for WAIS Index scores

|  | Sub-scale IQ score | | | |
| --- | --- | --- | --- | --- |
|  | **VCI** | **PRI** | **WMI** | **PSI** |
| Lifetime exposure to fluoridated water,  β (95%CI) |  |  |  |  |
| 100% | 2.90  (-2.13, 7.92) | 1.76 (-2.88, 6.41) | 1.37 (-2.98, 5.71) | -1.15 (-5.89, 3.58) |
| >0 -<100% | 5.67 (-0.03, 11.36) | -0.11 (-5.37, 5.16) | 4.69 (-0.23, 9.61) | -2.10  (-7.48, 3.27) |
| 0% | Ref | Ref | Ref | Ref |
|  |  |  |  |  |
| Dental fluorosis β (95%CI) |  |  |  |  |
| Yes | 0.40  (-3.62, 4.42) | 0.45  (-3.25, 4.16) | 1.46 (-2.09, 5.01) | 1.84 (-1.94, 5.62) |
| No | Ref | Ref | Ref | Ref |

**VCI**: Verbal comprehension index.

**PRI**: Perceptual reasoning index.

**WMI**: Working memory index.

**PSI**: Processing speed index.
Multivariable regression models controlling for socioeconomic factors, neurodevelopmental diagnosis, age at IQ test, breastfeeding duration, and toothbrushing frequency at age two years.

**Multiple imputation analysis**

A number of covariates had missing data. Multiple imputation method was applied to the dataset. SAS PROC MI was used with 10 iterations. The outcome variables were not imputed.

Similar to the main analysis, SAS PROC GLM was applied to the datasets using the MI_FILE statement to synthesise the estimates of each of the 10 iterations. Estimates of the analysis are present in Table 8 and 9.

**Table 8:** Estimates of the generalised linear multivariable regression model for full-scale IQ scores with percent lifetime exposure to fluoridated water

|  | FSIQ (N=357) | |
| --- | --- | --- |
|  | β | (95%CI) |
| % Lifetime exposure to fluoridated water (% LEFW) | 0.01 | (-0.03, 0.04) |
| Household income | 0.68 | (-1.27, 2.62) |
| Parental education | 3.95 | (1.24, 6.65) |
| Sex | 0.21 | (-2.45, 2.88) |
| Residential location | 0.59 | (-1.72, 2.90) |
| Neurodevelopmental diagnosis | -1.56 | (-3.37, 0.26) |
| Breastfeeding | 0.64 | (-0.37, 1.64) |
| Toothbrushing | 0.34 | (-2.36, 3.04) |

Multivariable regression models controlling for socioeconomic factors, neurodevelopmental diagnosis, age at IQ test, breastfeeding duration, and toothbrushing frequency at age two years.

**Table 9:** Estimates of the generalised linear multivariable regression model for full-scale IQ scores with dental fluorosis as exposure

|  | FSIQ (N=357) | |
| --- | --- | --- |
|  | β | (95%CI) |
| Dental fluorosis | 0.19 | (-1.92, 2.29) |
| Household income | 0.87 | (-1.09, 2.83) |
| Parental education | 4.62 | (1.95, 7.30) |
| Sex | -0.07 | (-2.77, 2.61) |
| Residential location | 0.72 | (-1.62, 3.05) |
| Neurodevelopmental diagnosis | -1.25 | (-3.07, 0.58) |
| Breastfeeding | 0.66 | (-0.36, 1.67) |
| Toothbrushing | 0.80 | (-1.94, 3.54) |

Multivariable regression models controlling for socioeconomic factors, neurodevelopmental diagnosis, age at IQ test, breastfeeding duration, and toothbrushing frequency at age two years.

**Imputation for missing information on the exposures to fluoride**

There were 12 participants with missing data on %LEFW, which were not imputed for the main analysis. 16 others were diagnosed with non-fluorotic lesions. For the analysis of this study, we applied the precautionary principle. We had decided not to include those cases as not having fluorosis as they had enamel discolouration (even not related to fluoride). Hence, we considered those non-fluorotic cases as missing in the main analysis. Sensitivity analysis was conducted to investigate potential impact of missing exposures on the study findings. First, all those who had completed the WAIS-IV but had missing exposure to fluoride were assigned to fully exposed (100%LEFW or having dental fluorosis) and then the same multivariable regression model 2 was generated for each primary outcome (Table 10, column 1). Those participants were then assigned to not-exposed (0%LEFW or no fluorosis) and then the same multivariable regression model 2 was generated for each primary outcome (Table 10, column 2). The generated estimates have not changed the study overall findings.

**Table 10:** Sensitivity analysis to investigate potential effect of missing exposures: Multivariable generalised linear regression models for full scale IQ scores

|  | FSIQ | |
| --- | --- | --- |
|  | **All missing cases assigned to full exposure** | **All missing cases assigned to no exposure** |
| % Lifetime exposure to fluoridated water (% LEFW), β (95%CI) |  |  |
| 100% | 0.63 (-3.31, 4.47) | 1.67 (-2.18, 5.53) |
| >0%-<100% | 2.47 (-1.98, 6.93) | 3.23 (-1.13, 7.61) |
| 0% | Ref | Ref |
|  |  |  |
| Dental fluorosis, β (95%CI) |  |  |
| Yes | -0.67 (-3.77, 2.42) | 0.43 (-2.87, 3.73) |
| No | Ref | Ref |
|  |  |  |

Models were adjusted for all covariates as in Model 2 of the main analysis.
